# Simple annealing process for producing unique one-dimensional fullerene crystal named fullerene finned-micropillar

**DOI:** 10.1038/s41598-020-76252-6

**Published:** 2020-11-06

**Authors:** Taku Onishi, Takahiro Tsukamoto, Takahide Oya

**Affiliations:** 1 Graduate School of Engineering Science, Yokohama National University, Yokohama, 240-8501 Japan; 2 Graduate School of Informatics and Engineering, The University of Electro-Communications, Chofu, 182-8585 Japan

**Keywords:** Electronic devices, Carbon nanotubes and fullerenes

## Abstract

We propose a process for easily fabricating a unique one-dimensional fullerene crystal, i.e., a fullerene finned-micropillar (FFMP). To fabricate a one-dimensional fullerene crystal more easily than when using current processes, fullerene was first annealed at 1173 K for 1 h with an argon gas flow of 0.5 L/min. We then examined how the FFMP structure changed when the fabrication process conditions, such as temperature, annealing time, and argon gas flow rate, were varied. FFMPs can be prepared within a short time and may have the same electrical characteristics as other one-dimensional crystals, e.g., fullerene nanowhiskers, so they are expected to be very useful for field-effect transistors, organic photovoltaics, and so on in the near future.

## Introduction

Fullerene^1^ is a spherical molecule composed of carbon atoms and is expected to be applied in many devices, such as field-effect transistors^2^, solar cells^3^, superconductive materials^4^, and chemical sensors^5^, because it has excellent properties such as resistance to molecular breakage, chemical reactions to produce derivatives, polymerization by light exposure, and n-type semiconducting properties. In addition, various one-dimensional crystals of fullerenes e.g., fullerene nanowhiskers (FNWs)^6^ and fullerene nanotubes (FNTs)^7^, have been reported. These crystals are considered to improve the performance of fullerene. However, fabricating one-dimensional fullerene crystals is laborious and time-consuming. For example, liquid–liquid interfacial precipitation (LLIP) is a widely known method for producing FNWs, but it requires expert skills and takes several days^8^. These requirements are considered factors that will hinder applying one-dimensional fullerene crystals in the future.

We propose a process for fabricating a new fullerene one-dimensional crystal named a fullerene finned-micropillar (FFMP) that is less complicated and time-consuming. This process will lead to mass production of one-dimensional fullerene crystals and has the potential to contribute to the further development of fullerene applications.

## Results

**Figure 1:**
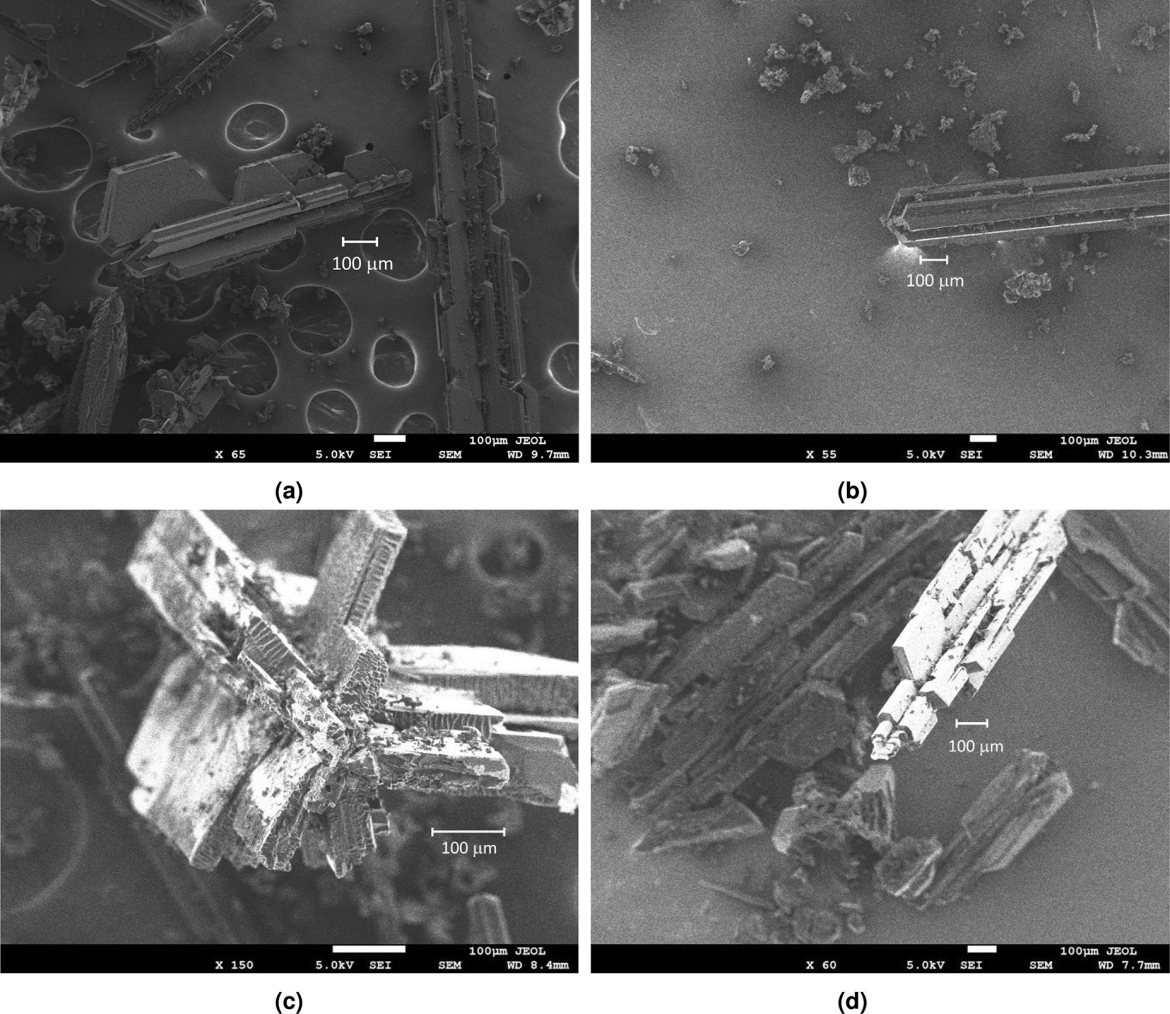
SEM images of samples; (**a**) and (**b**) side images, (**c**) tip image, and (**d**) oblique image.

We conducted a series of experiments to evaluate the effectiveness of our proposed process of fabricating FFMPs as described in “Methods” section. Scanning electron microscopy (SEM) images of the fabricated samples that were annealed at 1173 K for 1 h with an argon gas flow of 0.5 L/min are shown in Fig. 1. The samples had one-dimensional shape, i.e., a diameter of 100–200 microns, a length of 1 mm or more, and a hexagonal pillar or rectangular parallelepiped configuration as their axes. Furthermore, hexagonal or rectangular flakes like fins were attached to their surfaces. We used 1 g of fullerene as the initial amount (see the “Methods” section for more details about the experimental setup). After fabrication, we confirmed that a certain amount of fullerene remained at the put position before annealing and a certain amount of recrystallized material, including FFMPs and soot, was produced near the disposal line (the unheated section). The amount of these materials was almost the same as that of the remaining fullerene. If a small amount of fullerene, e.g., 1 mg, is used, an FFMP cannot be fabricated because a certain amount of fullerene must be decomposed by heat or flowed out. Therefore, we estimated that the yield of FFMPs should be around 40%, the remaining fullerene at the initial position should also be 40%, and the decomposed or flowed fullerene should be 20%.

**Figure 2:**
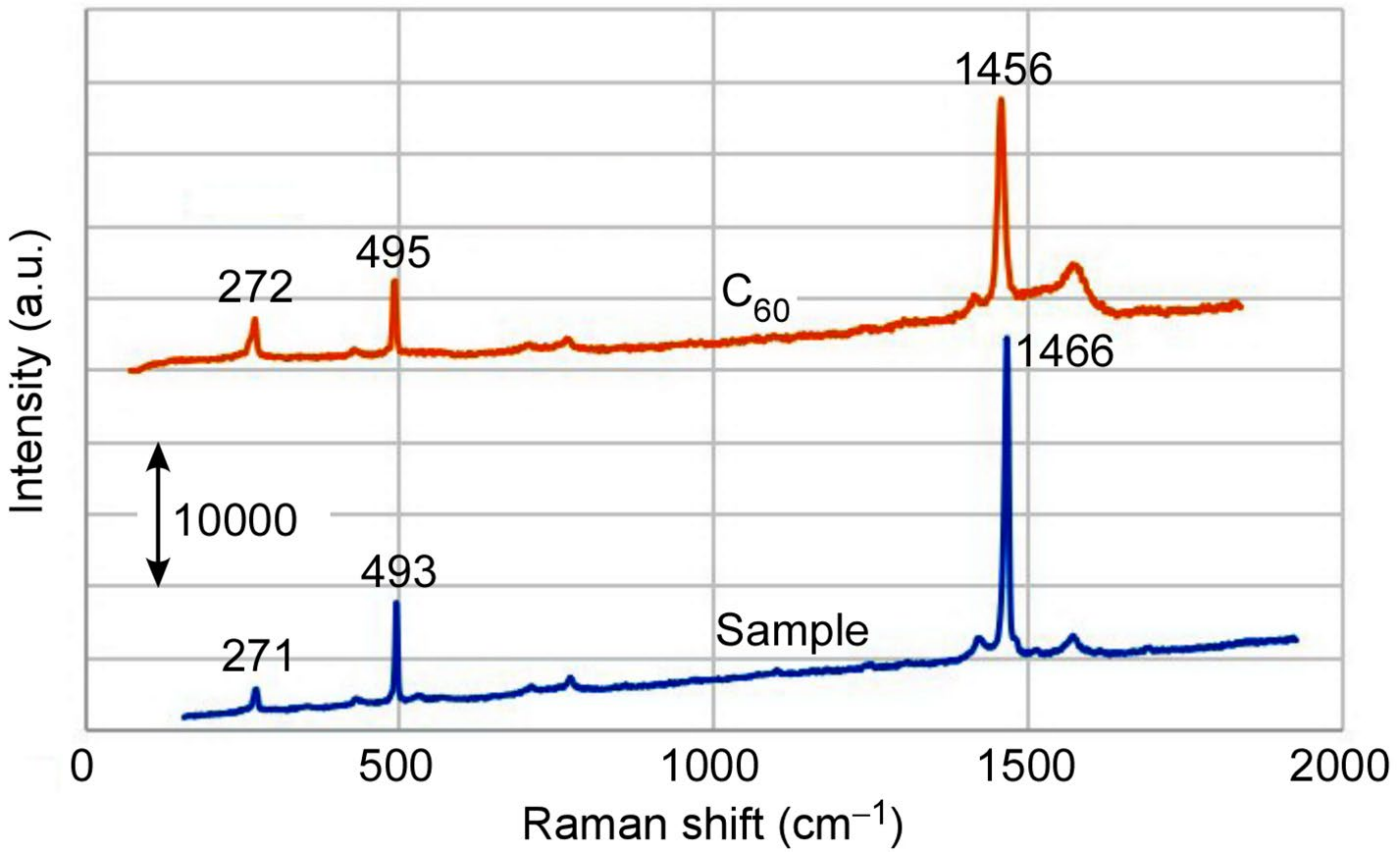
Raman spectra of sample and ${\hbox{C}}_{{60}}$ fullerene.

**Figure 3:**
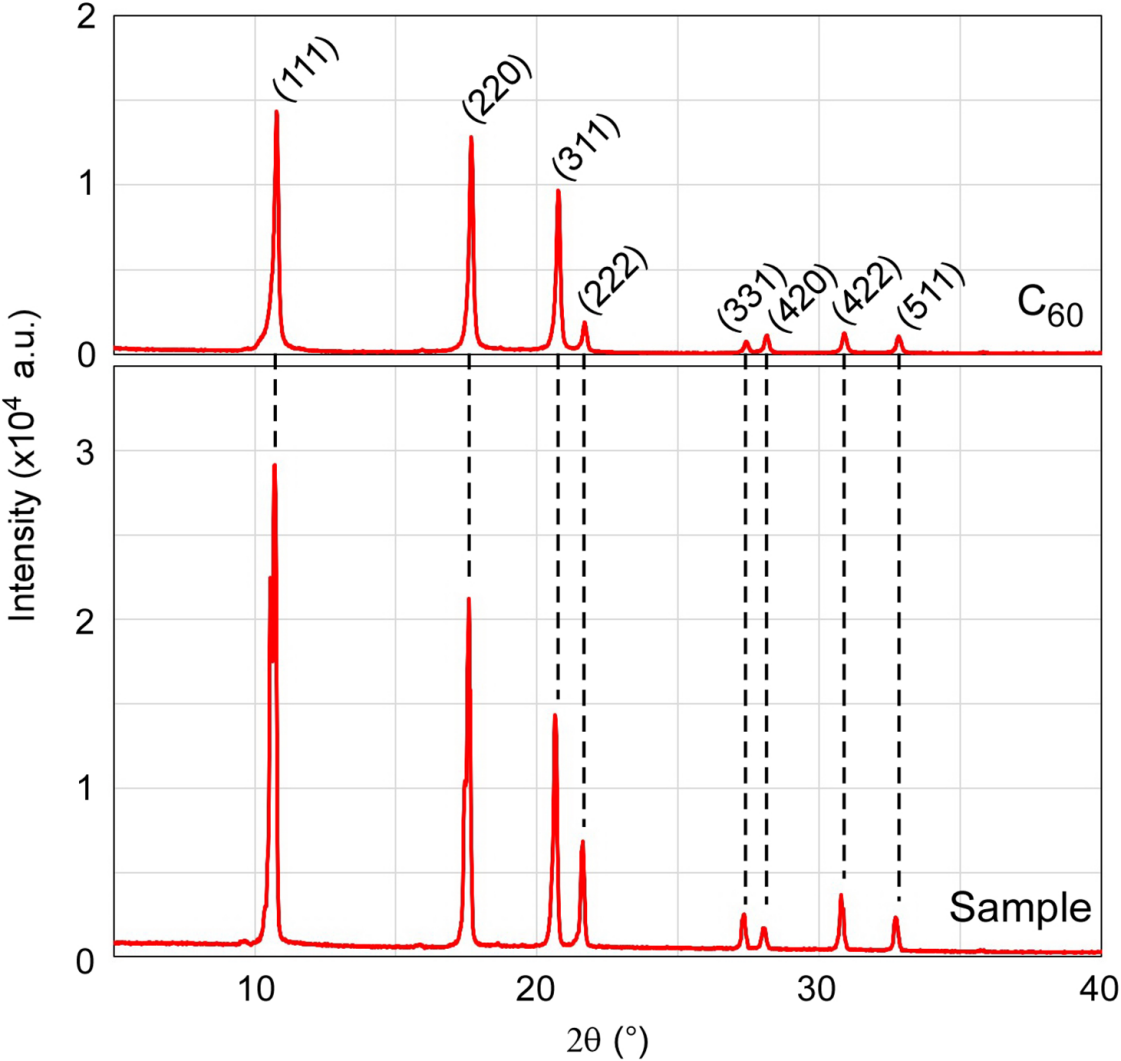
XRD spectra of sample and ${\hbox{C}}_{{60}}$ fullerene.

**Figure 4:**
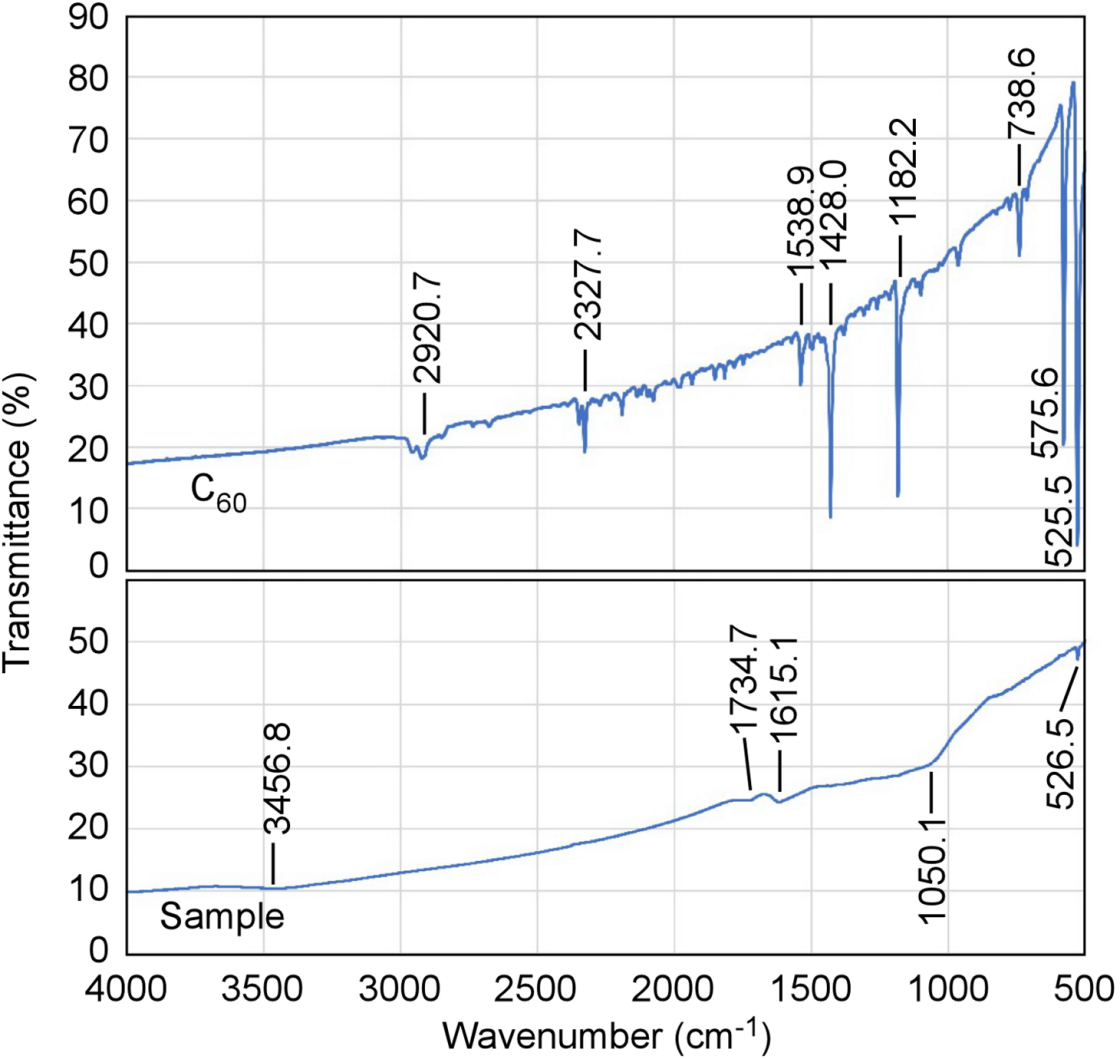
FTIR spectra of sample and ${\hbox{C}}_{{60}}$ fullerene.

**Figure 5:**
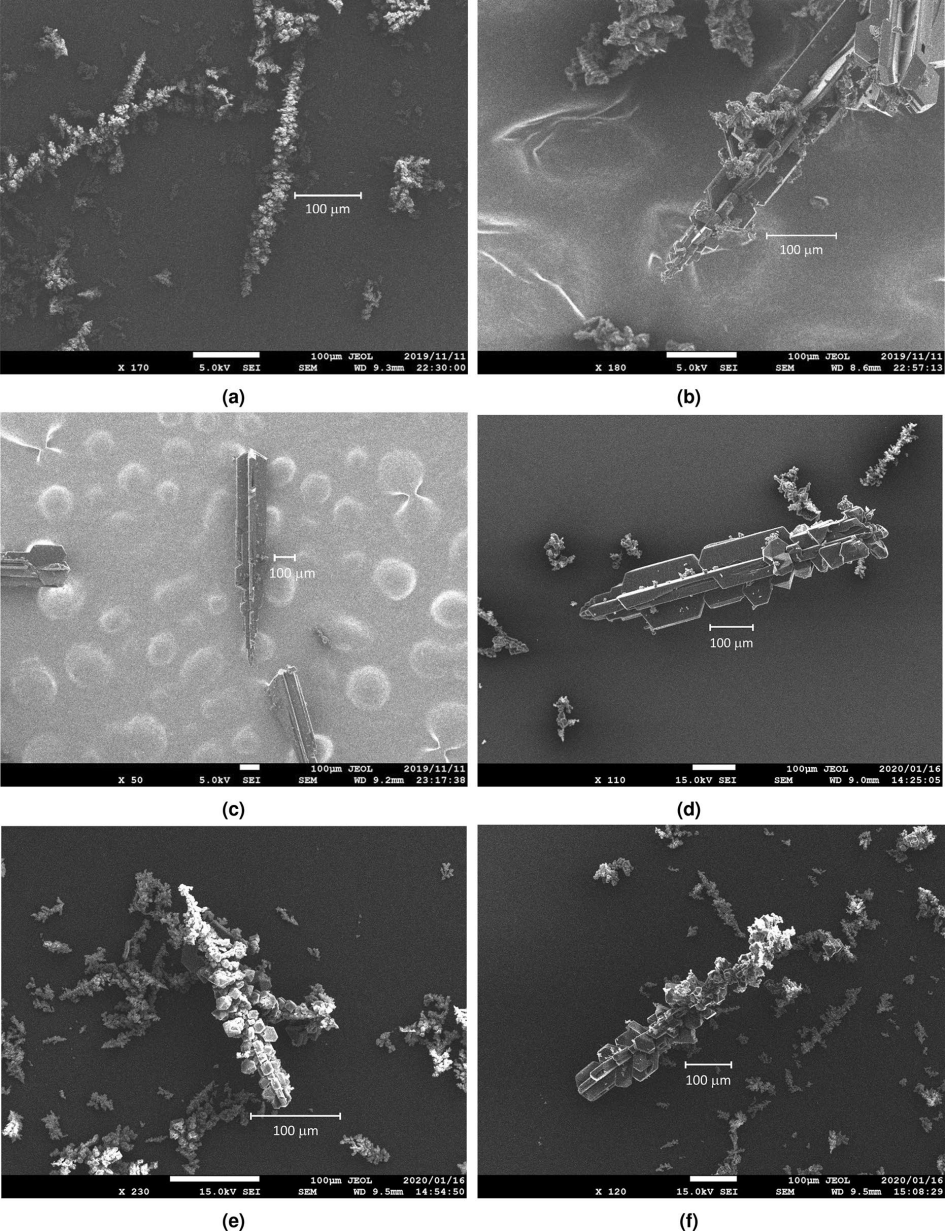
SEM images of samples prepared under various conditions. (**a**) annealed at 973 K. (**b**) annealed at 1073 K. (**c**) annealed for 10 min. (**d**) annealed with argon gas flow of 0.25 L/min. (**e**) and (**f**) annealed with argon gas flow of 1.0 L/min.

**Figure 6:**
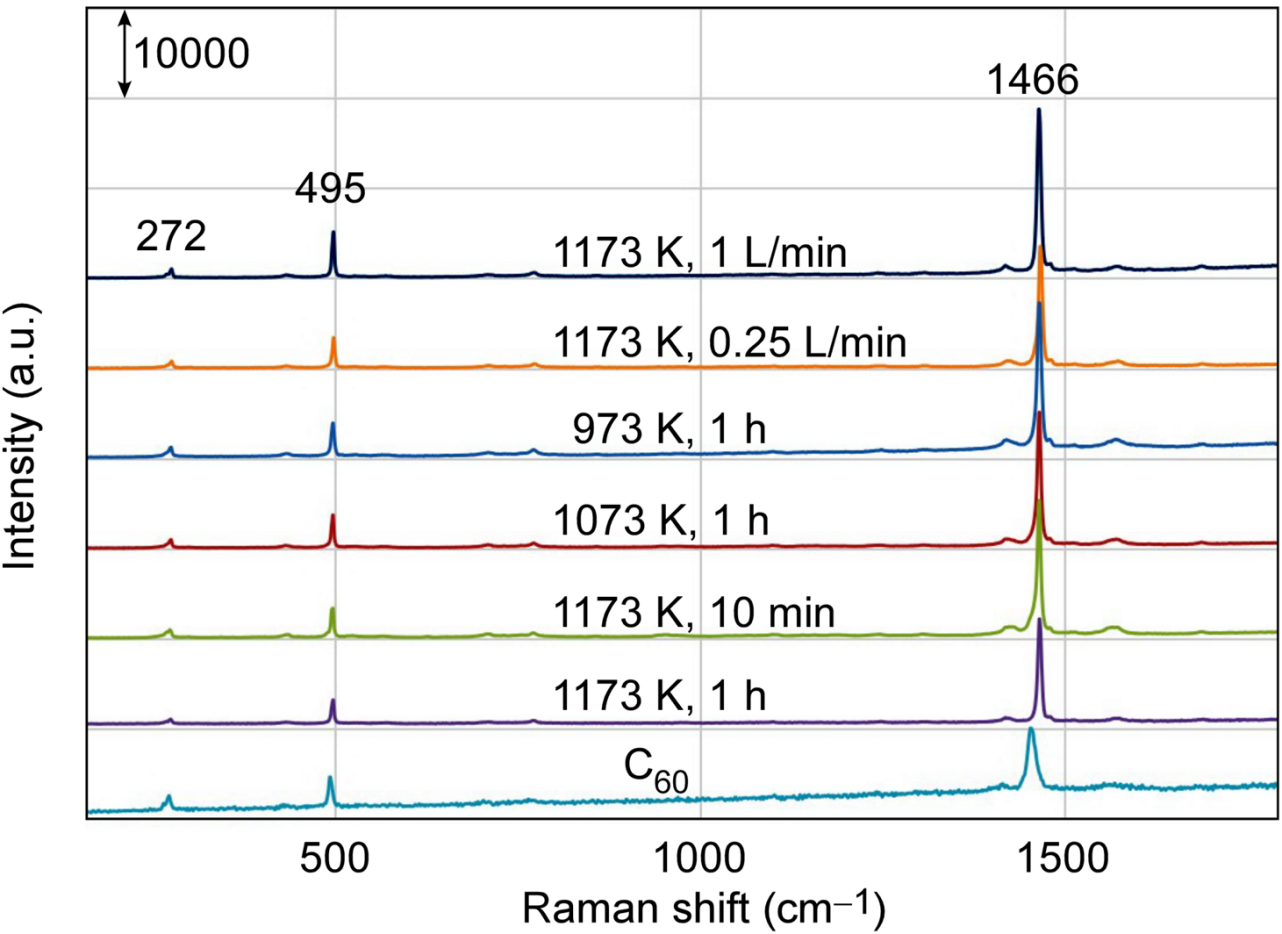
Raman spectra of samples prepared under various conditions.

The Raman spectrum of one of the samples is shown in Fig. 2. The sample had the same waveforms as ${\hbox{C}}_{{60}}$ fullerene, so the samples were definitely composed of ${\hbox{C}}_{{60}}$.

The results of X-ray diffraction (XRD) measurements shown in Fig. 3 confirm this unique crystal structure. The shape of the graph is similar to that of a fullerene crystal that has an fcc structure^9^. Therefore, we confirmed that the FFMPs have an fcc structure as well.

The results of Fourier transform infrared (FTIR) spectroscopy measurements are shown in Fig. 4. Here, the samples were mounted between potassium bromide (KBr) plates for the measurement. From the results, the FFMP seems to have some of features of polyhydroxylated fullerenes^10^, although the curve showed broad peaks and the peak positions did not entirely coincide with the result reported in Ref.^10^.

**Figure 7:**
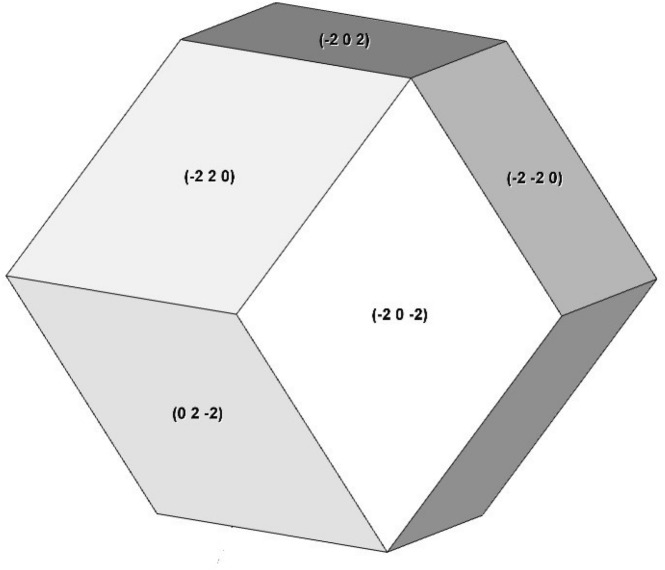
Solid composed of surfaces with only (220) reflections. Every vertex is intersection of 3 or 4 surfaces.

**Figure 8:**
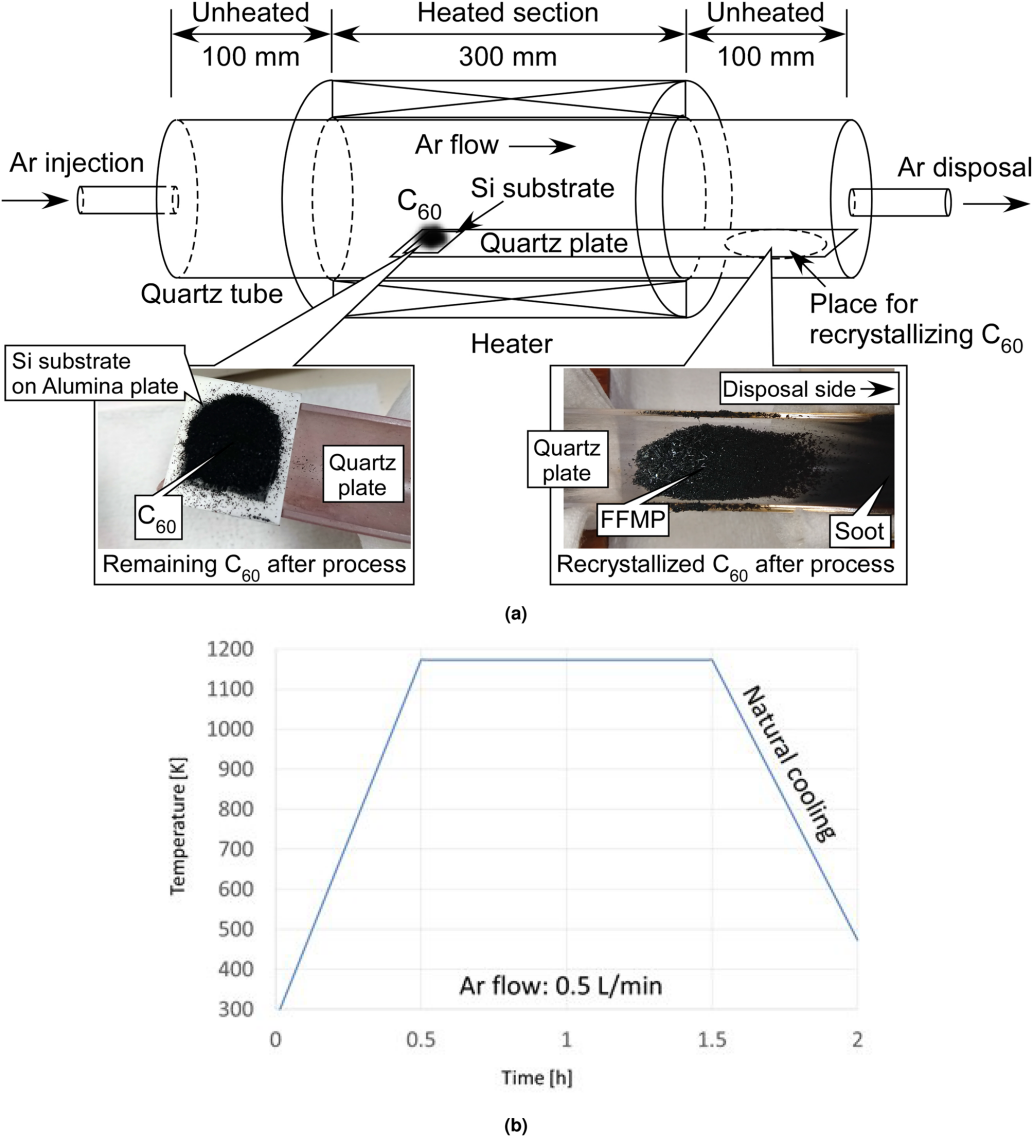
(**a**) Schematic of heating apparatus. Quarts plate (gutter-like curved-plate) is held in near center of quartz tube so as not to contact tube. Edge of plate is placed at center of heated section. Photos show remaining and recrystallized ${\hbox{C}}_{{60}}$ fullerene after proposed process. (Annealing condition was 1173 K for 1 h.) (**b**) Temperature during annealing process.

We then conducted experiments on samples under different conditions. Figure 5 shows SEM images of these samples. An FFMP could not be fully fabricated when the temperature was 973 K or the argon gas flow was 1.0 L/min. When the argon gas flow was 1.0 L/min, however, a sample in which the FFMP portion and non-FFMP portion were connected was obtained. From Fig. 6, all these samples were composed of ${\hbox{C}}_{{60}}$, i.e., they were recrystallized one-dimensional fullerene crystals and FFMPs.

## Discussion

A report on the nucleation of ${\hbox{C}}_{{60}}$ fullerene in the gas phase indicated that temperature significantly affects the crystal state^11^. It also indicated that only small crystal nuclei of ${\hbox{C}}_{{60}}$ molecules are randomly formed when the temperature is below 900 K and that the crystal nuclei of ${\hbox{C}}_{{60}}$ with long-range order are formed only at a temperature above 900 K. However, in our experiment, a substance in which small crystal nuclei randomly formed at 973 K, and FFMPs with long-range order nuclei formed only at 1073 K or more. Although these results seem to differ from those of the above report^11^, they are not considered unusual because argon gas was flowed through the apparatus in our study, and other gas molecules were not considered in the above report. At a specific temperature, ${\hbox{C}}_{{60}}$ molecules flocculate to form long-range order crystal nuclei, but argon molecules will hinder this process. As a result of this obstruction, the temperature required for producing ${\hbox{C}}_{{60}}$ crystal nuclei with long-range order increases. Therefore, FFMPs did not form at 973 K.

We failed to prepare FFMPs at not only 973 K but also with an increased flow rate of argon of 1.0 L/min. There are two possible common causes of these failures. The first is low temperature. In the experiment conducted with an argon flow rate of 1.0 L/min, it is conceivable that the sublimated ${\hbox{C}}_{{60}}$ molecules escaped from the heated section of the apparatus before it was sufficiently heated. As described above, crystals including FFMPs cannot be formed if the temperature is low. Instead, a collection of small grain crystals that are unlike FFMPs are produced.

The second cause is the low density of the sublimated ${\hbox{C}}_{{60}}$ molecules. When ${\hbox{C}}_{{60}}$ molecules were heated at 973 K, the efficiency of the sublimation of the ${\hbox{C}}_{{60}}$ molecules was low, and their density decreased when heated at a higher temperature. Furthermore, in the experiment conducted with the flow rate of argon of 1.0 L/min, the density of sublimated ${\hbox{C}}_{{60}}$ molecules was probably half that at the flow rate of 0.5 L/min. It is considered to be better to fabricate small crystal nuclei when the density of the sublimated ${\hbox{C}}_{{60}}$ molecules is low because it would be difficult to flocculate ${\hbox{C}}_{{60}}$ molecules and prepare large nuclei.

At this stage in our research, the initial small grains that enable the fabrication of the crystal nuclei of FFMPs could not be observed. A fabricated sample must be collected at the beginning or middle of the process. To meet this requirement, we must use SEM combined with a heating apparatus, i.e., *in site* observation is required. In the next stage, we will do this by modifying the proposed process and the devices for FFMP fabrication.

Although we clarified conditions for fabricating FFMPs, it is still uncertain why such unusual shapes are formed. To clarify this, simulations of crystal formation in air with argon molecules will be necessary. Furthermore, to simulate how FFMPs are fabricated, it may be necessary to adjust their shape, thickness, and length.

FFMPs have intersections of 3 or 4 fullerene plates at their ends. The numbers 3 or 4, not 2 or 5, are explained in terms of the Miller index. The XRD spectrum shown in Fig. 3 show mainly (111), (220), and (311) reflections. Among these reflections, only the (220) reflection has intersections of 3 and 4 surfaces, as shown in Fig. 7. Therefore, if the side surfaces of the fullerene plates consist of the (220) reflections, this may explain why FFMPs have vertexes of 3 or 4 plates.

Although the (220) reflection has been described, the peak of the (111) reflection is the highest of the various peaks of the XRD graph of the sample, as shown in Fig. 3. From the graph, it is considered that the surfaces of the fullerene plates principally consist of the (111) reflection. Furthermore, the peak of the (311) reflection is the lowest of the three reflections. It seems that the surfaces of FFMPs consist of (111) and (220) reflections, and the peaks of the other reflections in the XRD graph are not from the surfaces but from inside the plates. In the above experiment, additional shoulder peaks within (111) and (220) appeared. It is not clear what these additional shoulder peaks are. We think that they are caused by the distinctive finned structure of FFMPs.

In addition, although almost all FTIR spectrum obtained from FFMPs was broad and the peak positions did not entirely coincide with the data of polyhydroxylated fullerene reported in Ref.^10^, FFMP seems to have features of it partly. From this, there is a possibility that a little water might be adsorbed on the used ${\hbox{C}}_{{60}}$ fullerene and it might influence ${\hbox{C}}_{{60}}$ fullerene under the process. We will investigate their origin and unknown points in the next stage of our research. We used a Raman microscope, X-Ray diffractometer, and Fourier transform infrared spectroscopy for characterization in this study. However, unknown points remain as described above. For clearer discussion, e.g., X-ray photoelectron spectroscopy (XPS) experiment must be conducted.

FFMPs may have conductivity similar to that of FNWs because the crystal structure is the same fcc structure as in FNWs^12^. FNWs have high electrical conductivity and n-type semiconductor functionality^13^. Of course, the crystal shapes and sizes of FNWs and FFMPs slightly differ, but similar electrical characteristics can be expected. Fullerene Finned-“Nano”pillars (FFNPs) are expected to be fabricated by modifying the proposed process. For this, we will continue to find suitable conditions.

Although there are some unknown factors, FFMPs can be prepared quickly and may have the same electrical characteristics as FNWs, so we believe that FFMPs will be useful for field-effect transistors, organic photovoltaics, and so on in the near future.

## Methods

The experiment was conducted using a small heating apparatus (GFA430VN, THERMO RIKO Co., Ltd.). The internal structure of the device is shown in Fig. 8a. In this experiment, only ${\hbox{C}}_{{60}}$ fullerene (prepared by Sigma-Aldrich, 98%) was used. First, 1 g of ${\hbox{C}}_{{60}}$ fullerene was set on a silicon substrate. This substrate on an alumina plate was put the edge of the gutter-like quartz curved-plate. The edge was placed at the center of the heated section. The ${\hbox{C}}_{{60}}$ fullerene was then annealed at 1173 K for 1 h by following our set heating process, as shown in Fig. 8b, to sublimate the fullerene. During annealing, to prevent the fullerene from oxidizing, 0.5 L of argon per minute was flowed through the apparatus to remove the oxygen in the air. The apparatus was heated only at the center and was not heated in the vicinity of the argon injection and disposal sections. The sublimated ${\hbox{C}}_{{60}}$ fullerene in the heated section was recrystallized near the argon disposal (unheated) section. After the annealing, recrystallized fullerene crystals were collected. The photos in Fig. 8a show the remaining and recrystallized fullerene that formed using the proposed process.

After that experiment, the annealing conditions were changed, and other crystals were prepared. First, the annealing temperature was changed to 1073 and 973 K. Second, the annealing time was changed to 10 minutes. Third, the flow rate of argon was changed to 0.25 and 1.0 L/min. We observed the materials obtained under these conditions.

For evaluation of obtained samples, we used a scanning electron microscope (JSM-7001F, JEOL Ltd.) for imaging and a Raman microscope (inVia Reflex, Renishaw plc.), X-Ray diffractometer (Ultima IV, Rigaku corporation), and Fourier transform infrared spectroscopy (FT-IR 6200, JASCO corporation) for characterization.
